# Progress and Prospects in Human Genetic Research into Age-Related Hearing Impairment

**DOI:** 10.1155/2014/390601

**Published:** 2014-07-22

**Authors:** Yasue Uchida, Saiko Sugiura, Michihiko Sone, Hiromi Ueda, Tsutomu Nakashima

**Affiliations:** ^1^Department of Otolaryngology, Aichi Medical University, Nagakute, Aichi 480-1195, Japan; ^2^Department of Otorhinolaryngology, National Center for Geriatrics and Gerontology, Aichi 474-8511, Japan; ^3^Department of Otorhinolaryngology, Nagoya University Graduate School of Medicine, Nagoya 466-8550, Japan

## Abstract

Age-related hearing impairment (ARHI) is a complex, multifactorial disorder that is attributable to confounding intrinsic and extrinsic factors. The degree of impairment shows substantial variation between individuals, as is also observed in the senescence of other functions. This individual variation would seem to refute the stereotypical view that hearing deterioration with age is inevitable and may indicate that there is ample scope for preventive intervention. Genetic predisposition could account for a sizable proportion of interindividual variation. Over the past decade or so, tremendous progress has been made through research into the genetics of various forms of hearing impairment, including ARHI and our knowledge of the complex mechanisms of auditory function has increased substantially. Here, we give an overview of recent investigations aimed at identifying the genetic risk factors involved in ARHI and of what we currently know about its pathophysiology. This review is divided into the following sections: (i) genes causing monogenic hearing impairment with phenotypic similarities to ARHI; (ii) genes involved in oxidative stress, biologic stress responses, and mitochondrial dysfunction; and (iii) candidate genes for senescence, other geriatric diseases, and neurodegeneration. Progress and prospects in genetic research are discussed.

## 1. Introduction

Age-related hearing impairment (ARHI), also known as presbycusis, is the most common sensory impairment among the elderly. Typical features of ARHI are gradual progression later in life and bilaterally symmetrical sensorineural hearing loss which involves the higher frequencies. However, ARHI shows extensive variation, as is also observed in the senescence of other functions. The progression and the severity of hearing impairment vary considerably between individuals. ARHI is a complex, multifactorial trait that is attributable to confounding intrinsic and extrinsic factors. Genetic predisposition could account for a sizable proportion of interindividual variation. Since we published our review on the molecular genetic epidemiology of ARHI in 2011 [1], the number of investigations into the genetics of ARHI has grown hugely. Here, we present the most recent findings in genetic research and discuss the progress of such research to date and future prospects.

This review is divided into the following three areas, although there is likely some overlap between the genes described in each:

- genes causing monogenic hearing impairment with phenotypic similarities to ARHI,
- genes involved in oxidative stress, biologic stress responses, and mitochondrial dysfunction,
- candidate genes for senescence, other geriatric diseases, and neurodegeneration.

Recent topic-based genetic contributions, which have been followed by a flurry of additional reports, have been preferentially selected.

## 2. Genes Causing Monogenic Hearing Impairment with Phenotypic Similarities to ARHI 

These genes, which can cause hearing impairment such as pronounced progressive sensorineural hearing loss in the high frequencies, are excellent candidate ARHI susceptibility genes [2]. Many investigators have attempted to verify the association between certain genes causing monogenic nonsyndromic hearing impairment and ARHI. We provide leading examples here and also list them in Table 1.

### 2.1. DFNA28 =* GRHL2*/*TFCP2L3*/*BOM *(OMIM: 608576)

The DFNA28 (OMIM: 608641) locus is occupied by the* GRHL2* (*grainyhead like 2)* gene, which is also known as* TFCP2L3* (*transcription factor cellular promoter 2-like 3*) or* BOM* (*brother of mammalian grainyhead*), and is a widely expressed transcription factor in human epithelial tissues [11].* GRHL2* spans approximately 177 kb on chromosome 8q22.3 (NCBI 37/hg19), contains 16 exons, and is translated into a 625-amino-acid protein. It was first associated with the* DFNA28* locus through mapping studies involving a five-generation North American family affected with mild to moderate postlingual progressive bilateral sensorineural hearing loss [12]. A frameshift mutation in* GRHL2 *(c.1609_1610insC) that results from a truncation in exon 14 is responsible for DFNA28-associated autosomal dominant hearing loss.

Very recently, a second DFNA28-causing mutation and the first splice-site mutation in* GRHL2* (c.1258-1G>A) were reported in a family affected with nonsyndromic hearing loss [12, 13]. GRHL2 participates in the differentiation and maintenance of epithelial cells throughout life [11]. Impaired epithelial cell integrity is the most reasonable pathologic explanation as to its involvement in late-onset hearing impairment [3, 12]. van Laer et al. have concluded that* GRHL2* is an ARHI susceptibility gene from the results of an association study performed with 2418 samples from individuals with ARHI at nine centers in seven European countries [3]. After statistical analysis of 703 single nucleotide polymorphisms (SNPs) selected from 70 candidate genes, they demonstrated that the three top-ranked SNPs all resided in* GRHL2*, in a region of approximately 16 kb. Particularly, the most significant SNP, rs10955255, was replicated in two of nine independent sample sets; and the odds ratios (ORs) of all nine sample sets had similar implications. However, when Lin et al. tried to replicate these results in a Han Taiwanese population, they found no positive association between the* GRHL2* SNP locus (rs10955255: A/G) in intron 1 (coordinate: 102605581) and ARHI [4].

The hearing loss in the family with c.1609-1610insC can be categorized as mild to moderate across all frequencies in the initial stages but progressing toward severe hearing loss of the high frequencies in the fifth decade. The age at onset is variable; the youngest patient was diagnosed in his first decade. DFNA28 hearing loss does not then entirely match the typical features of ARHI. The functional properties of GRHL2 within the inner ear will also critically depend on the presence of its target genes, given that GRHL2 is a transcription factor [3].

### 2.2. DFNA2 =* KCNQ4* (OMIM: 603537)

*KCNQ4* (*potassium voltage-gated channel member 4*) encodes a voltage-gated potassium channel [14], which plays a role in potassium recycling in the inner ear and is expressed in hair cells of the cochlea and the vestibular apparatus and in the auditory nuclei of the brainstem [15]. Signal transduction in the cochlea depends on maintenance of a high potassium concentration and a positive potential in the endolymph. This is mediated through ion transport across the stria vascularis into the hair cells and efflux of K^+^ probably through voltage-gated ion channels. Mutations in these channels have been thought to implicate candidate genes for individuals with slowly progressive hearing loss.

Mutations in* KCNQ4* cause an autosomal-dominant type of nonsyndromic hearing loss, DFNA2. It has been reported that DFNA2-associated hearing loss is typically late in onset, is involved in high-frequency hearing loss, and progresses over time [16]. Although the* KCNQ4* gene has 14 exons encoding a protein of 695 amino acids, the mutations seem to cluster around exons 4–7, with missense mutations being the most common [17]. The progressive nature of the hearing loss is a common feature of patients with* KCNQ4* mutations, regardless of the particular mutation [18].

van Eyken et al. examined SNPs spread across* KCNQ4* for association with ARHI by treating ARHI as a quantitative trait in two independent Caucasian populations. For both populations, several SNPs in a region spanning 13 kb in the middle of the* KCNQ4* gene were significantly associated with ARHI [5].

### 2.3. DFNA5 =* DFNA5 *(OMIM: 608798)

DFNA5 was originally discovered in a Dutch family with autosomal dominant nonsyndromic hearing impairment [19]. The auditory feature of DFNA5 closely resembles that of the most frequent type of ARHI, namely, sensorineural progressive loss that begins in the high frequencies. van Laer et al. have investigated quantitative trait linkage analysis (a quantitative measure of high-frequency hearing loss) in extended pedigrees as well as in two different association studies involving two amino acid substitution SNPs in* DFNA5*. They have concluded that no strong association exists between* DFNA5* and ARHI, because no significant linkage was detected between ARHI and microsatellite markers from the* DFNA5* region and no significant differences were found in genotypes between individuals with good hearing and those with hearing impairment [6].

Different mutations have been found among Korean, Chinese, and Dutch families with DFNA5-associated hearing loss, all exerting a highly specific gain-of-function effect. In these families exon 8 is skipped during splicing, causing a frameshift mutation and premature termination of the protein [20, 21]. Later, it became clear that the DFNA5 protein acts as a tumor suppressor in several frequent types of cancer and that it can induce apoptosis [22].

Apoptosis is a key contributor to the development of presbycusis or ARHI. In a review article focusing on the programmed death of the hair cell, Op de Beeck et al. discussed* DFNA5 *as one of several mutations in apoptosis genes that cause monogenic deafness [23]. They suggested that apoptosis contributes not only to the pathology of acquired forms of hearing impairment, but also to genetic hearing impairment.

### 2.4. DFNA9 =* COCH* (OMIM: 603196)

The autosomal dominant nonsyndromic hearing disorder DFNA9 is characterized by late onset bilateral progressive high-frequency sensorineural hearing impairment with associated variable vestibular dysfunction and starts to manifest between 20 and 50 years of age [24]. Its features are similar to those of ARHI but hearing deterioration with DFNA9 occurs at a younger age. Point mutations in the* COCH* (*cochlin* or* coagulation factor C homology*) gene, encoding the protein product, cochlin, are responsible for DFNA9-associated hearing loss.* COCH* mutations have been found in individuals living on four continents and the possibility that they play an important role in presbycusis and disorders of balance has been considered [25]. Phenotypic comparisons among families with different DFNA9-causing mutations in the* COCH *gene have been reported, with individuals presenting in the 95th percentile of the air conduction threshold levels for presbycusis for a given age and sex according to ISO 7029 [24, 26]. In a study on the association between* COCH*-variant T352S (rs 1045644) and ARHI, no significant effect of the SNP on quantitative trait values for ARHI was found [7].

### 2.5. DFNB15/DFNB72/DFNB95 =* GIPC3 *(OMIM: 608792)

Autosomal recessive deafness 15 (DFNB15), also known as DFNB72 or DFNB95, is caused by homozygous mutations in the* GIPC3* (*GAIP interacting protein 3, C terminus*) gene on chromosome 19p13.3.

Charizopoulou et al. identified a sequence polymorphism in the PDZ domain of Gipc3 as the cause of progressive sensorineural hearing loss (age-related hearing loss 5,* ahl5*) and audiogenic seizure susceptibility (*jams1*) in BLSW mice and they screened panels of families of Dutch and Indian origin presenting with autosomal recessive nonsyndromic hearing impairment to examine whether mutations in* GIPC3* underlie human sensorineural hearing loss [8]. They identified the* ahl5*/*jams1* and DFNB15/DFNB95 loci and revealed a function for Gipc3 in peripheral auditory signal transmission in mice and humans. They proposed that* GIPC3* and its paralogs are excellent candidate genes for ARHI.

Susceptibility to metabotropic glutamate receptor 7 (mGluR7) or glutamate metabotropic receptor 7 (GRM7) excitotoxicity has been suggested as a risk factor for ARHI [27–29]. Charizopoulou et al. [8] noted that mGluR7 and Gipc3 show identical localization domains in the mouse cochlea and also share functional similarities, quoting studies of Friedman et al. [27] and Yi et al. [30]. Rehman et al. described one frameshift and six missense mutations in* GIPC3* that cosegregate with DFNB72 associated with mild to profound hearing loss in six large families; their data provide statistically significant evidence for genetic linkage [9].

### 2.6. DFNA22/DFNB37 =* MYO6* (OMIM: 600970)

*MYO6* encodes myosin VI, an unconventional actin-based motor protein, expressed in the hair cells of the inner ear that is necessary for maintaining their normal stereociliar structure [31, 32].* MYO6* has been reported to be responsible for hearing loss associated with loci DFNA22 and DFNB37 [33, 34].

Oonk et al. compared the detailed audiometric characteristics of a large Dutch family with autosomal dominant hearing loss caused by a mutation in* MYO6* with those of three different DFNA22 families published previously [10]. Hearing loss in the family closely resembled presbycusis and the authors hypothesized that rare variants of* MYO6* may contribute to presbycusis.

## 3. Genes Involved in Oxidative Stress, Biologic Stress Responses, and Mitochondrial Dysfunction

Oxidative stress plays a major role in the overall aging process. Reactive oxygen species (ROS), such as superoxide anion, hydrogen peroxide, and hydroxyl radicals, are generated as byproducts of mitochondrial oxidative phosphorylation. Overproduction of free radicals and ROS results in damage to mitochondrial DNA (mtDNA). The mitochondrial “vicious cycle” theory of aging proposes that accumulation of mtDNA mutations may lead to enhanced mitochondrial ROS production and subsequent increased oxidative stress during aging [35].

Free radicals, ROS, and associated mitochondrial dysfunction are causes of age-related neurosensory hearing loss. Complex enzymatic and nonenzymatic systems act mutually to counteract ROS-mediated oxidative damage. Oxidative stress in the inner ear, secondary to the decrease in defense mechanisms caused by certain polymorphisms related to a battery of human antioxidant systems, could make individuals more susceptible to ARHI. A number of researchers have focused on several genes and loci involved in oxidative stress and have identified mitochondrial DNA mutations as candidate genes for the development of ARHI [36].

Two classes of antioxidant enzymes are active in the cochlea: enzymes involved in glutathione (GSH) metabolism (e.g., glutathione S-transferase, GST; glutathione peroxidase, GPX1; and glutathione reductase, GSR) and enzymes involved in the breakdown of superoxide anions and hydrogen peroxide (e.g., catalase, CAT, and Cu/Zn superoxide dismutase, SOD1) [37–40].

### 3.1. *NAT2* (OMIM: 612182), GST Family Genes

N-Acetyltransferase enzymes are responsible for the detoxification of large numbers of exogenic substrates and are important for balance of oxidative status. Two N-acetyltransferase isozymes, NAT1 and NAT2, have been identified in humans. A consensus nomenclature for the N-acetyltransferase genes has been established, with NAT1*4 and NAT2*4 designated as the reference alleles for* NAT1* (*N-acetyltransferase 1*) and* NAT2 *(*N-acetyltransferase 2*), respectively [41, 42]. An international nomenclature committee publishes updates in* NAT1 *and* NAT2* alleles at the website http://nat.mbg.duth.gr. Variant* NAT2* alleles or haplotypes possessing combinations of SNPs are segregated into clusters possessing a signature SNP either alone or in combination with others. Common human* NAT2* alleles (haplotypes) discussed in the previous studies regarding ARHI are listed (see Table 2).

Ünal et al. investigated associations between ARHI and the* NAT* variations NAT2*5A, NAT2*6A, NAT2*7A/B, and NAT2*14A [44]. They studied a population of 68 white individuals of Turkish descent with presbycusis and found that the risk for presbycusis was 15.2-fold higher among individuals with the mutant allele NAT2*6A than among those with a wild-type genotype (*P* = .013).

The human glutathione S-transferase (GST) family is composed of at least 8 classes (alpha, A; kappa, K; mu, M; omega, O; Pi, P; sigma, S; theta, T; and zeta, Z) with multiple subfamilies per class [45, 46]. GST enzymes play a key role in antioxidant defenses and a high level of GST enzymes is important to protect DNA from damage. Some classes, such as* GSTM1* (mu, chromosome 1p13.3),* GSTP1* (pi, chromosome 11q13), and* GSTT1* (theta, chromosome 22q11.2), are polymorphic. The* GSTT1* polymorphism is caused by deletion of a substantial part of the gene, causing a reduction of GSTT1 activity in all tissues, whereas in individuals homozygous for the* GSTM1* deletion the enzyme is absent [45].

Angeli et al. investigated the association among audiometric patterns and polymorphisms of the antioxidant enzymes GSTT1, GSTM1, and NAT2 in a hospital-based cohort study of adults with ARHI [37]. Participants with mutant alleles for* GSTT1 *were more likely to have a “high-frequency steeply sloping” audiogram than participants with the wild-type genotype, suggesting that the basal turn of the cochlea is susceptible to GSTT1-regulated oxidative stress.

van Eyken et al. investigated an association between ARHI and genes related to oxidative stress, including polymorphisms in single genes (GSTM1, GSTT1, NAT2*5A, NAT2*6A, and NAT2*7A) with a large set of 2,111 independent samples from a general European population and a Finnish population [43]. They observed significant associations between ARHI and NAT2*6A in the European population but no association between* GSTM1* or* GSTT1*. However, in the Finnish population they found significant associations between ARHI and both genes.

Bared et al. investigated the association of different polymorphisms of* GSTM1*,* GSTT1*, or* NAT2* with ARHI in 55 affected adults and 79 controls with normal hearing and showed an increased risk for ARHI among white adults carrying the* GSTM1* and the* GSTT1 *null genotypes and the NAT*6A mutant allele [38]. Genotyping of the* NAT2 *polymorphisms (NAT25*A, NAT2*6A, NAT2*6B, NAT2*7A, NAT2*7B, NAT2*14A, and NAT2*14B) was performed. The frequency of the NAT2*6A mutant genotype (heterozygous and null genotype combined) was more frequent among patients with presbycusis (60%) than in controls (34%; *P* = 0.0086, OR = 2.88, 95% confidence interval [95% CI] = 1.355–6.141). The* GSTT1 *null genotype was present in 34% of controls and in 60% of white patients with ARHI (*P* = 0.0067, OR = 2.843, 95% CI = 1.379–5.860). The* GSTM1* null genotype was more frequent in patients with ARHI.

### 3.2. mtDNA Mutations

Wallace and Fan have divided the mtDNA variants clinically relevant to disease susceptibility into three classes: recent deleterious mutations resulting in maternally transmitted disease, ancient adaptive variants that predispose individuals to disease in different environments, and the age-related accumulation of somatic mtDNA mutations that erode function and provide the aging clock [47]. These mutations can be inherited (constitutional) or acquired (somatic) and show a particularly poor genotype-phenotype correlation. Different mutations in the same gene causing different phenotypes and the same mutation at different levels of heteroplasmy causing different phenotypes result in the extraordinary clinical variability of mtDNA-associated diseases.

mtDNA mutations can exist as point mutations, nucleotide insertions or deletions, and large deletions. mtDNA deletions were reported to accumulate with age in a variety of tissues [48]. Analyses of human temporal bones indicated that the mtDNA 4977-bp deletion, the so-called “common” deletion, occurred frequently in ARHI patients [49, 50]. Markaryan et al. used a real-time polymerase chain reaction assay to quantify the mtDNA in archival cochlear tissue samples derived from temporal bone and found a significant association between the common deletion level in human cochlear tissue and the severity of hearing loss [51].

mtDNA polymorphisms typically reflect different ethnic backgrounds. Specific mtDNA polymorphisms have now been classified into a number of mitochondrial haplogroups. Estimated worldwide haplotype frequencies (Mitomap: http://www.mitomap.org/) show that there are 20 recognized mtDNA haplogroups within the European community, 18 in the Asian community and 10 in the African community. Some of these haplotype groups have been associated with ARHI. Manwaring et al. found that haplogroups U and K associated independently with a higher prevalence of ARHI [52]. However, recently, Bonneux et al. found no significant associations of inherited common and rare coding variants, their mutation load, or European haplogroups with ARHI. They concluded that inherited mitochondrial variants are not a major factor in ARHI [53].

## 4. Candidate Genes for Senescence, Other Geriatric Diseases, and Neurodegeneration

### 4.1. *APOE* (OMIM: 107741)

The* APOE* (*Apolipoprotein E*) gene encodes apolipoprotein E (ApoE), a multifunctional protein of the lipid and lipoprotein transport system involved mainly in metabolism of dietary lipids. The three major isoforms, human ApoE2, -E3, and -E4, are encoded by three common alleles of* APOE* called the *ε*2, *ε*3, and *ε*4 alleles. These alleles represent haplotypes of two SNPs in the coding region of* APOE*. The *ε*2 allele encodes a cysteine (Cys) at amino acid position 112 and at position 158. Allele *ε*3 encodes a Cys at 112 and an arginine (Arg) at 158, while the *ε*4 allele encodes an Arg at 112 and at 158 [54]. These polymorphisms within the* APOE* gene were strongly associated with late-onset Alzheimer disease. The *ε*4 allele is the ancestral and high-risk form, the *ε*3 allele is the most common in humans and the neutral allele, and the *ε*2 allele is associated with decreased risk for Alzheimer disease.

O'Grady et al. found that, compared with the general population, the* APOE* *ε*4 allele was less prevalent in a study population of 89 individuals (median age; 64 years) with nonsyndromic adult-onset sensorineural hearing loss, although they originally assumed that the *ε*4 allele might predispose individuals to sensorineural hearing loss and ischemic injury in the cochlea by causing atherosclerotic vascular disease [55]. Recently, a converse finding that the presence of the* APOE* *ε*4 allele was associated with ARHI overall frequency was demonstrated in the Leiden 85-plus study, a population-based study involving 85-year-olds [56]. The* APOE* *ε*4 allele was associated with a 2.0-fold increased risk for hearing impairment (95% CI, 1.0–4.0).

### 4.2. *MTHFR* (OMIM: 607093)

Folate metabolism is essential for cellular functioning. Polymorphisms in genes encoding critical enzymes in folate metabolism play important and interrelated roles in the pathophysiology of numerous disorders such as cancer and cardiocerebrovascular disease. The* MTHFR* (*methylenetetrahydrofolate reductase*) C677T polymorphism (rs1801133) is one of the most extensively investigated functional polymorphisms. It results in an alanine-to-valine substitution and a 30% decrease in MTHFR enzyme activity in heterozygotes and a 60% decrease in homozygotes [57].

The* MTHFR* T allele has been shown to be associated with an increased risk for sudden sensorineural hearing loss in several different studies [58–60]. Interestingly, an inverse association between the same variant and ARHI was also demonstrated and may be due to a protective effect in individuals carrying the variant with a sufficient dietary supply of folate [61, 62]. We investigated the effects of the* methionine synthase* (*MTR*) A2756G and* MTHFR* C677T gene polymorphisms on the risk for hearing impairment in community-dwelling Japanese adults aged 40–84 years who participated in the National Institute for Longevity Sciences, Longitudinal Study of Aging (NILS-LSA), an ongoing population-based biennial study. Cumulative data (5,167 samples in the accumulated total) were analyzed with generalized estimating equations. The* MTHFR* 677T allele was significantly associated with a reduced risk for hearing impairment only when the participants were wild-type homozygotes for* MTR* A2756G (per-T allele OR of* MTHFR* for development hearing impairment in the* MTR* AA genotype: 0.7609 [95% CI: 0.6178–0.9372]). In addition, a subgroup analysis demonstrated that the favorable effect of the* MTHFR* 677T allele on the risk of developing hearing impairment was independent of serum folate and plasma total homocysteine levels. We speculated that the* MTHFR* 677T allele has a potentially advantageous effect of preventing imbalances in the nucleotide pool during DNA synthesis, ensuring that DNA replication occurs with high fidelity.

### 4.3. *EDN1* (OMIM: 131240)

Endothelin-1, which is known to be an extremely potent vasoconstrictor peptide, is a prominent endothelial mediator. It is widely distributed throughout the mammalian body in cardiovascular and noncardiovascular tissues, including the nervous system, and is also expressed in the spiral modiolar artery, endolymphatic sac, vestibule, stria vascularis, and spiral ganglion cells [63, 64].

We have reported an association between the Lys198Asn (G/T) polymorphism (rs5370) in the* EDN1* (*endothelin-1*) gene and hearing impairment in the aforementioned NILS-LSA participants aged 40 years to 79 years at baseline [65]. Mutant T-allele carriers were demonstrated to have a higher risk for hearing impairment than homozygotic carriers of the wild-type allele in several models that adjust for different combinations of confounding variables.

### 4.4. *UCP2* (OMIM: 601693)

Uncoupling proteins (UCPs) are a family of mitochondrial proton transporters and may be regulators of thermal control and energy metabolism. UCPs are thought to be activated by superoxide and considered to decrease generation of mitochondrial free radicals.

We reported a relationship between gene polymorphisms in* UCP1* (*uncoupling protein 1*) and* UCP2* (*uncoupling protein 2*) and hearing impairment in the aforementioned NILS-LSA [66]. Data were analyzed to assess the effects of* UCP1* A-3826G and* UCP2* Ala55Val polymorphisms on hearing impairment. The* UCP1* A3826G polymorphism did not exhibit any significant association with ARHI; however, the* UCP2* Ala55Val polymorphism did show a significant association with hearing impairment under the dominant, recessive, and additive models (*P* < 0.05).

### 4.5. Neurotransmitter-Related Genes,* GRM7* (OMIM: 604101) and* GRM8* (OMIM: 601116)

GRM7 (glutamate receptor, metabotropic, 7) is thought to be central to maintaining glutamate synaptic transmission and homeostasis in the mammalian cochlea at the synapses between hair cells and the dendrites of afferent auditory nerve fibers [28].

Friedman et al. identified and subsequently validated a significant risk association between the* GRM7* locus and ARHI [27]. In a study of three independent sample groups identified on the basis of a pooling genome-wide association study (GWAS), three genetic factors were described: a single SNP (rs11928865) and two haplotypes (haplotype block 6 consisting of SNPs rs11928865 and rs9877154, and haplotype block 7 consisting of SNPs rs6804466, rs3828472, rs12497688, rs9819783, and rs11920109). The authors demonstrated that the protein product, mGluR7, was expressed in inner and outer hair cells and in the spiral ganglion nerve cell bodies of the inner ear of mice and humans.* GRM7 *mRNA expression was also present in the neurons of the spiral ganglion in the mouse cochlea.

Newman et al. have delved further into the effect of* GRM7* on ARHI [28]. They performed mixed modeling analyses in a European-American population from Rochester in the United States to explore the relationship between* GRM7* haplotypes (haplotype blocks 6 and 7) and SNP genotypes and various measures of auditory perception, such as the free-field hearing-in-noise test (HINT) and suprathreshold gap detection tasks. They showed that* GRM7* alleles were associated primarily with peripheral measures of hearing loss and particularly with speech detection in older adults.

Luo et al. conducted a case-control candidate gene association study in an elderly male Han Chinese population composed of 982 men with ARHI and 324 controls with normal hearing [29]. The results of the study suggest that* GRM7* SNP rs11928865 (TT genotype) occurs more frequently in ARHI patients with certain audiogram-shape subtypes of ARHI, namely, sloping-shaped and 2–4 kHz abrupt loss-shaped phenotype patterns.

New candidate loci were reported from a GWAS for hearing quantitative traits among participants recruited from several isolated villages within the international consortium called G-EAR [67]. Eight primary loci were detected in a series of genes expressed within the inner ear, including* GRM8 *(*glutamate receptor, metabotropic, 8*), and the candidate genes located in positive GWAS regions belonged to several different gene families, which overlap only insensibly with those already identified as causing hearing impairments.

### 4.6. *ESRRG* (OMIM: 602969)

Estrogen has recently been identified as a novel modulator of hearing function [68, 69]. There is now considerable evidence linking estrogen signaling, the estrogen receptors, and estrogen-related receptors (ESRR) with auditory protection [68, 70].

Nolan et al. conducted an investigation involving a total of 6134 individuals in three independent cohorts: (i) the 1958 British birth cohort; (ii) a London ARHL case-control cohort; and (iii) a cohort from isolated populations of Italy and Silk Road countries to evaluate whether variants in estrogen signaling genes may be risk factors for adult-onset hearing loss. The rs2818964 SNP in the* ESRRG* (*estrogen-related receptor, gamma*) gene was associated with hearing status in the London ARHL cohort, the minor allele being associated with poorer hearing but only in women. This association was replicated in the Carlantino cohort and the combined cohort of isolated populations from Italy and Silk Road countries, both in the direction of the allelic effect and in the female-specific association [71]. In line with these findings, ESRRG was shown to be expressed in the adult mouse inner ear and hearing assessment in* Esrrg *knock-out mice revealed worse impairment in female mice (−/−) than in male mice (−/−). These results indicate that ESRRG plays a role in maintenance of hearing in both humans and mice.

## 5. Future Prospects

The degree of ARHI varies substantially among individuals, as is also observed in senescence of other functions. It is not always true that deterioration of hearing with age is inevitable. During the last 10 years or so, tremendous progress has been made through human genetic research into hearing impairment and our understanding of the complex mechanisms of hearing has increased greatly. It might be that certain environmental risk factors are potentially detrimental in a limited number of individuals only, depending on their genetic background. It is hopeful that successful preventive intervention customized according to knowledge of genetic susceptibility will help to preserve good hearing even in the oldest old.

## Figures and Tables

**Table 1:** Recent reports regarding association between ARHI and monogenic forms of hearing impairment.

Genes causing monogenic hearing impairment with phenotypic similarities to ARHI						
Genes	Monogenic nonsyndromic hearing impairment	Authors	Reference	Publication year	Object region or ancestry of the study subjects	Remarks
*GRHL2/TFCP2L3/BOM *(OMIM: 608576)	DFNA28	van Laer et al.	[3]	2008	9 centers in 7 European countries	Authors have concluded that *GRHL2 *is an ARHI susceptibility gene.
		Lin et al.	[4]	2011	Taiwan	No positive association was found.

*KCNQ4* (OMIM: 603537)	DFNA2	van Eyken et al.	[5]	2006	Netherlands and Belgium	Several SNPs in this gene were significantly associated with ARHI.

*DFNA5 *(OMIM: 608798)	DFNA5	van Laer et al.	[6]	2002	Framingham in USA	Authors have concluded that no strong association exists between *DFNA5* and ARHI.

*COCH *(OMIM: 603196)	DFNA9	Fransen et al.	[7]	2004	Belgium and Netherlands	No significant effect of the SNP for ARHI was found.

*GIPC3 *(OMIM: 608792)	DFNB15/DFNB72/DFNB95	Charizopoulou et al.	[8]	2011	Families of Dutch and Indian origin	A missense mutation of *Gipc3* was associated with age-related hearing loss 5 (*ahl5*) in mice and point mutations of human *GIPC3* were found in hearing loss cases segregating in two small human families.
		Rehman et al.	[9]	2011	Pakistani families	Authors reported seven mutations of *GIPC3 *associated with mild to profound hearing loss segregating in seven large families.

*MYO6* (OMIM: 600970)	DFNA22/DFNB37	Oonk et al.	[10]	2013	Dutch family	The audiological findings in the Dutch DFNA22 family supported the hypothesis that the phenotype of the specific *MYO6* mutation mimics presbycusis.

**Table 2:** Common human* NAT2* alleles (haplotypes) discussed in the previous studies regarding ARHI (modified from Hein, 2006 [41]).

Allele (haplotype)^1^	Nucleotide change(s)^2^	Amino acid change(s)^3^	Catalytic activity^4^	Results in the previous ARHI studies
*NAT2* ∗ *4 *	None	None	High	Wild type

***NAT2*** ∗ ***5A***	341T>C (rs1801280); 481 C>T	I114T	Low	Negative by Angeli et al. [37], van Eyken et al. [43], Ünal et al. [44], and Bared et al. [38]

***NAT2*** ∗ ***6A***	282 C>T; 590 G>A (rs1799930)	R197Q	Low	Negative by Angeli et al. [37]
				Positive by Ünal et al. [44], van Eyken et al. [43], Bared et al. [38]

***NAT2*** ∗ ***6B***	590 G>A (rs1799930)	R197Q	Low	Negative by Angeli et al. [37] and Bared et al. [38]

***NAT2*** ∗ ***7A***	857 G>A	G286E	Low	Negative by van Eyken et al. [43]
				(*NAT2*∗*7A/B* combined) Negative by Ünal et al. [44]

***NAT2*** ∗ ***7B***	282 C>T; 857 G>A (rs1799931)	G286E	Low	Negative by Bared et al. [38]

***NAT2*** ∗ ***14A***	191 G>A (rs1801279)	R64Q	Low	Negative by Ünal et al. [44], Bared et al. [38]
				Data not shown by van Eyken et al. [43]

***NAT2*** ∗ ***14B***	191 G>A (rs1801279); 282 C>T	R64Q	Low	Negative by Bared et al. [38]

^1^Common *NAT2* alleles (haplotypes) associated with low catalytic activity and slow acetylator phenotype are bolded.

^2^Signature SNP for each cluster is shown with underline.

^3^Amino-acid substitutions that confer reduced *NAT2* activities.

Individuals homozygous for alleles indicated by boldface in annotation 1 are slow acetylators.
